# Current Applications of Bacteriocin

**DOI:** 10.1155/2020/4374891

**Published:** 2020-11-03

**Authors:** Abebe Worku Negash, Berhanu Andualem Tsehai

**Affiliations:** ^1^Department of Biotechnology, College of Natural and Computational Science (CNCS), Adigrat University, P.O. Box 50, Adigrat, Ethiopia; ^2^Department of Biotechnology, Institute of Biotechnology (IoB), University of Gondar, P.O. Box 196, Gondar, Ethiopia

## Abstract

Bacteriocins are multifunctional, ribosomally produced, proteinaceous substances with pronounced antimicrobial activity at certain concentrations. They are produced by bacteria and certain members of archaea to inhibit the growth of similar or closely related bacterial strains. These molecules have antimicrobial activity against pathogenic and deteriorating bacteria, which justifies their biotechnological potential. They are classified into 3 major classes based on their structural and physicochemical properties: class I bacteriocin, class II bacteriocin, and class III bacteriocin. Bacteriocins inhibit the growth of target organisms by functioning primarily on the cell envelope and by affecting gene expression and protein production within cells. The use of bacteriocins has been reported for the following: food preservation, diverse therapeutic purposes such as treatment of peptic ulcer, spermicidal agent, and woman care, anticancerous agent, veterinary use, skincare, and oral care, and also for plant growth promotion in agriculture among others.

## 1. Introduction

All living organisms produce antimicrobial proteins (AMPs), many of which are called antimicrobial peptides because of their relatively small size. Bacteria produce two types of AMP: those which are synthesized by ribosomes (also called bacteriocins) and AMPs that are not synthesized by ribosomes, without structural genes coding for these AMPs [1].

In general, bacteriocins are multifunctional, ribosomally produced proteinaceous substances with pronounced antimicrobial activity at certain concentrations [1]. They are protein toxins produced by bacteria and certain members of archaea to inhibit the growth of similar or closely related bacterial strains [2]. These molecules have antimicrobial activity against pathogenic and deteriorating bacteria, justifying their biotechnological potential. If the bacteriocins produced by a bacterium inhibit other bacteria belonging to the same species, they are generally considered to be narrow-spectrum bacteriocins. In contrast, if they inhibit bacteria belonging to another genus, they are considered to be broad-spectrum bacteriocins. Interestingly, bacteriocin-producing bacterial cells are resistant to their antimicrobial peptides, which are mediated by specific immunity proteins produced by host cells [3]. The genes encoding bacteriocin production and immunity are generally organized in operon clusters and may reside on mobilizable elements such as chromosome in conjunction with transposons or on a plasmid [4].

Reports on the first bacteriocin production were from *Escherichia coli* in 1925, and this peptide was named “colicins” to reflect the microbial source [5]. However, bacteriocins produced by LAB are of particular importance, because these bacteria received GRAS status by the American Food and Drug Administration (FDA) [4]. Thus, nisin produced by *Lactococcus lactis* was the first bacteriocin to gain widespread commercial application. Since then, a large number of bacteriocins from a diverse group of bacterial strains have been identified. Bacteriocin production could be considered as advantageous to the producer as, insufficient amounts; these peptides can kill or inhibit bacteria competing for the same ecological niche or the same nutrient pool [6].

Bacteriocins have many positive properties that have made them particularly interesting for various applications. LAB bacteriocins are inherently tolerant of high thermal stress and are known for their activity over a wide pH range. These antimicrobial peptides are also colorless, odorless, and tasteless, which further improves their potential usability. They are also easily degraded by proteolytic enzymes due to their proteinaceous nature. Consequently, bacteriocin fragments do not live long in the human body or in the environment, which minimizes the chance of target strains interacting with degraded antibiotic fragments [7].

Bacteriocins can be considered “designer drugs” that target specific bacterial pathogens. *Escherichia coli* and other members of the *Enterobacteriaceae* family are the few examples of Gram-negative bacteria and lactic acid bacteria; *Bacillus* species belong to Gram-positive bacteria which produce bacteriocins [8].

The objective of this review is to summarize important information about bacteriocins, their classification, their diverse mechanism of action, and potential applications in the food industry, livestock industry, medicine, and agriculture.

## 2. Comparison between Bacteriocins and Antibiotics

When they are in comparison, bacteriocins have a ribosomally synthesized nature, while antibiotics are produced by multiple enzymes complexes. Often bacteriocins exhibit bactericidal or bacteriostatic effects on a narrow spectrum of bacteria, but traditional antibiotics have a wider spectrum. Besides, most bacteriocins are more effective against their target bacteria than antibiotics at lower concentrations [9]. Bacteriocins are often considered more natural because they are believed to have been present in many of the foods consumed since ancient times. Bacteriocins are inactivated by enzymes, such as trypsin and pepsin, found in the gastrointestinal tract and therefore do not alter the microbiota of the digestive tract [10].

## 3. Classification of Bacteriocins

### 3.1. Bacteriocins of Gram-Positive Bacteria

The classification of bacteriocins has been periodically reviewed. The latest classification organizes bacteriocins into three main classes based on their structural and physicochemical properties [4].

#### 3.1.1. Class I Bacteriocins/Lantibiotics

Lantibiotics are small (<5 kDa) heat-stable peptides that are highly posttranslational modified and that contain characteristic polycyclic thioether amino acids such as lanthionine, methyl-lanthionine, and unsaturated amino acids such as dehydroalanine and 2-amino isobutyric acid. Lantibiotics are further subdivided into two types, depending on the difference in charge [11]. Type A-lantibiotics such as nisin and lacticin 3147 are flexible screw-shaped molecules with a positive charge of 2–4 kDa which causes the formation of pores in the cell membrane of the target organism and thus lead to depolarization of the target species cytoplasmic membrane [11].

Type B-lantibiotics are peptides of 2–3 kDa without net charge or net negative charge. These are globular molecules that interfere with cellular enzymatic reactions such as cell wall synthesis. Mersacidin secreted by *Bacillus* spp. is an example of this type [12].

#### 3.1.2. Class II Bacteriocins

Class II-bacteriocins are small peptides (<10 kDa) heat-stable, containing no lanthionine, which are not modified after translation beyond the elimination of a leader peptide and the formation of a conserved N-terminal disulfide bridge. They have an amphiphilic helical structure, which allows them to insert into the membrane of the target cell, leading to depolarization and death [11].

Bacteriocins of subclass IIa such as pediocin PA-1 and sakacin A are monomers and have an N-terminal consensus sequence Tyr-Gly-Asn-Gly-Val-Xaa-Cys. They are active in particular against *Listeria monocytogenes* [11].

Bacteriocins in subclass IIb include lactacin F and lactococcin G. These are two-component bacteriocins, in which two distinct peptides act synergistically to generate an antimicrobial effect [11]. The third subclass IIc contains circular bacteriocins such as gassericin A, circularin A, and carnocyclin A [13]. These peptides carry two transmembrane segments that facilitate the formation of pores in the target cells [14].

#### 3.1.3. Class III Bacteriocins

Class III bacteriocins are heat-labile proteins with a high molecular weight (>30 kDa). Some of the colicins, megacins (from *Bacillus megaterium*), klebicin (from *Klebsiella pneumonia*), helveticin I (from *Lactobacillus helveticus*), and enterolysin (from *Enterococcus faecalis*) are members of this group [11].  Table 1 shows the summary of bacteriocin classification with few examples.

### 3.2. Bacteriocins of Gram-Negative Bacteria

The bacteriocins of Gram-negative bacteria are divided into two main groups: high molecular mass proteins (30–80 kDa) known as colicins and low molecular mass peptides (between 1 and 10 kDa) called microcins. Colicins are produced by strains of *Escherichia coli* that harbor a colicinogenic plasmid. Microcins are generally highly stable molecules, resistant to proteases, extreme pH values, and temperatures. They are produced by enteric bacteria under stress conditions, particularly nutrient depletion [15].

## 4. Bacteriocin Mode of Action

**Figure 1:**
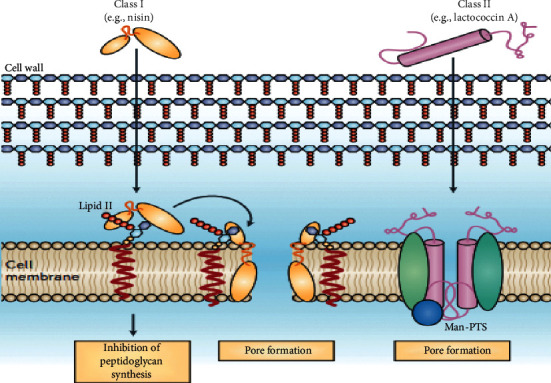
Mechanism of action of bacteriocins on Gram-positive bacteria [16].

Bacteriocins inhibit the growth of target organisms in various mechanisms. These mechanisms can be roughly subdivided into mechanisms that function primarily on the cell envelope and mechanisms that are primarily active in the cell that affect gene expression and protein production. Certain bacteriocins, and in particular many of those that inhibit Gram-positive bacteria, work by attacking the cell envelope. Certain class I bacteriocins inhibit lipid-II on the cell membrane, thus eliminating the synthesis of peptidoglycan. Other bacteriocins form pores to inhibit or kill their target bacteria (Figure 1). For example, class II bacteriocins such as lactococcin A bind to the pore-forming receptor mannose phosphotransferase system (Man-PTS) [16].

It has been shown that some members of class I or lantibiotic bacteriocins, such as nisin, have a dual mode of action. They can bind to lipid-II, the main transporter of peptidoglycan subunits from the cytoplasm to the cell wall, and therefore prevent correct cell wall synthesis, leading to cell death. Furthermore, they can use lipid-II as a docking molecule to initiate a process of membrane insertion and pore formation that leads to rapid cell death. A lantibiotic with two peptides, such as lacticin 3147, can have these dual activities distributed over two peptides, while mersacidin has only the lipid-II binding activity but does not form pores [13].

**Figure 2:**
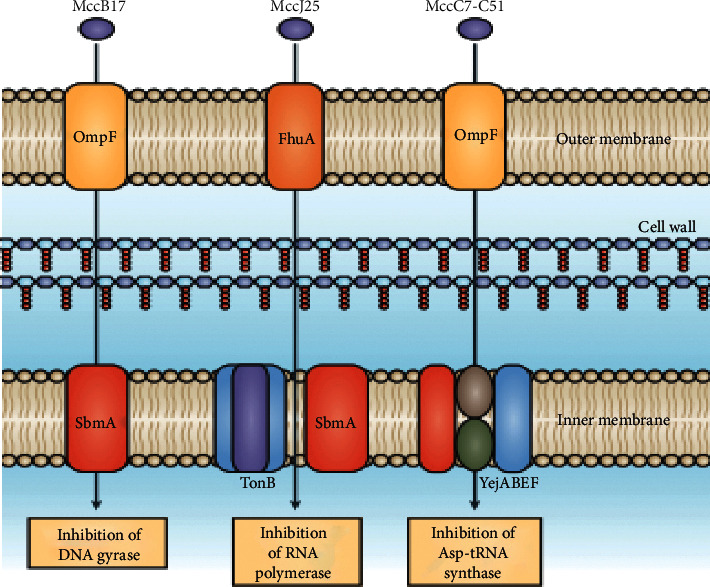
Mechanism of action of bacteriocins on Gram-negative bacteria [16].

Many bacteriocins that inhibit Gram-negative bacteria (and thus need to be transported through the outer and, in many cases, inner membranes before functioning) control their target bacteria by interfering with DNA, RNA, and protein metabolism. For instance, MccJ25 inhibits RNA polymerase, microcin B17 (MccB17) inhibits DNA gyrase, and MccC7-C51 inhibits aspartyl-tRNA synthetase. There are also exceptions, such as MccE492, which work by forming pores (Figure 2) [16].

Some bacteriocins show antimicrobial activity through their enzymatic activities. For example, colicin E2 shows DNase activity, colicin E3 shows RNase activity, and megacin A-216 shows phospholipase activity against the target organism [8].

In general, the class II peptides have an amphiphilic helical structure, which allows them to insert into the membrane of the target cell, leading to depolarization and death. On the contrary, large bacteriolytic proteins, such as lysostaphin (class III bacteriocins), can function directly on the cell wall of Gram-positive targets, leading to death and lysis of the target cell [13].

## 5. Application of Bacteriocins

Bacteriocins have different applications in the food industry, pharmaceutical industry, and agriculture.

### 5.1. Food Preservation

Bacteriocins have been used extensively in the preservation of food. The use of bacteriocins in the food industry has been extensively investigated, particularly in dairy products, eggs, vegetables, and meat products [4].

Nisin is used in over 48 countries and has FDA approval, and NisaplinTM is sold as a natural food protectant. It is effective in several food systems, inhibiting the growth of a wide range of Gram-positive bacteria, including many important food-borne pathogens, such as *Listeria monocytogenes*. It is mainly used in canned food and dairy products and is especially effective in use in the production of processed cheese and spreads where it protects against heat-resistant spore-forming organisms such as those of the genera *Bacillus* and *Clostridium*. This is particularly important in the case of the prevention of *Clostridium botulinum* infection as there can be serious consequences due to toxin formation by this species. Many bacteriocins still to be commercialized such as lacticin 3147 and lacticin 481, which have demonstrated the potential for exploitation as natural preservative and flavor enhancers [6].

Pediocin PA-1 is a broad-spectrum lactic acid bacteriocin that exhibits particularly strong activity against *Listeria monocytogenes* and is used as a food preservative [17].

There are at least three ways in which bacteriocins can be incorporated into a portion of food to improve its safety, namely, the use of a purified/ semipurified bacteriocin preparation as an ingredient in a food, by incorporating an ingredient previously fermented with a bacteriocin-producing strain or using a bacteriocin-producing culture to replace all or part of starter culture in fermented foods to produce bacteriocin in situ [6].

Bacteriocins can also be used to improve food quality and sensory properties, for example, increasing the rate of proteolysis or in the prevention of gas blowing defect in cheese. Another application of bacteriocins is bioactive packaging, a process that can protect the food from external contaminations, which improves food safety and shelf life [4].

### 5.2. Treatment of Peptic Ulcer

Peptic ulcers are caused by an imbalance between the defense mechanisms of the gastroduodenal mucosa and the damaging forces of gastric acid and pepsin, combined with overlapping lesions of environmental or immunological agents. Anaerobic *Helicobacter pylori* counts are excessive in patients with gastric and duodenal ulcers. Bacteriocin shows maximum inhibition against *H. pylori* that causes peptic ulcer disease. Based on the antimicrobial activity profiles, bacteriocins produced by *Pediococcus acidilactici* BA28 was proposed for the formulation of topical personal care therapies aimed at the prevention and treatment of many human diseases, in particular, peptic ulcers [18].

### 5.3. Spermicidal Activity and Woman Care

Bacteriocins are potential spermicidal agents because of their ability to affect sperm motility [19]. Fermenticin HV6b is a class IIa antimicrobial peptide produced by *Lactobacillus fermentum* HV6b MTCC 10770 isolated from the human vaginal ecosystem. It can inhibit the growth of bacteria, *Gardnerella vaginalis*, *Mobiluncus*, *Staphylococci*, and *Streptococci*, causing vaginal infections in humans. Fermenticin HV6b has a unique sperm immobilization and spermicidal activity. A new formulation with *Lactobacillus fermentum* HV6b or fermenticin HV6b can be used alone or in combination with the production of vaginal creams that can protect the human vagina against microbial infections and also act as contraception [20].

*Bacillus amyloliquefaciens* produce bacteriocin subtilosin, which inhibits the vaginal pathogen *Gardnerella vaginalis* but is not a healthy normal vaginal microbiota and the vaginal cell surface itself. Different concentrations (28.3–113.3 *μ*g/ml) of subtilosin were tested which could significantly reduce the motility of human spermatozoa. Together with the antimicrobial property, subtilosin has been shown to eliminate the motility and forward progression of human spermatozoa in a dose-dependent manner and can, therefore, be considered a common spermicide. Subtilosin would, therefore, be a valuable component in topical personal care products focused on contraception and BV prophylaxis and treatment [21].

### 5.4. Anticancerous Activity

The potential use of bacteriocins in cancer therapy is due to its inhibition of the synthesis of DNA and membrane proteins, which causes apoptosis or cytotoxicity in tumor cells [19]. A bacteriocin nisin may serve as a new potential therapeutic for the treatment of head and neck squamous cell carcinoma (HNSCC), as it induces preferential apoptosis, cell cycle stops, and reduces cell proliferation in HNSCC cells, compared to primary keratinocytes [22].

A natural variant of nisin (nisin ZP; 95%, high-content) has been reported for its antitumor effects in vitro and in vivo. Nisin ZP induced the highest level of apoptosis in HNSCC cells compared to low-content nisin (2.5%). HNSCC cells treated with increasing concentrations of nisin ZP exhibited increasing levels of apoptosis and decreasing levels of cell proliferation, clonogenic capacity, and sphere formation. Therefore, nisin ZP exhibits greater antitumor effects than low-content nisin and, therefore, has the potential to serve as a novel therapeutic for HNSCC [23].

Another important bacteriocin of *Enterococcus mundtii* strain C4L10 could be used as a possible antimicrobial agent and for antiproliferative agents in cancer control. Cancer cell lines, such as HSC3 oral cancer, MCF7 breast cancer, H1299 lung cancer, and HCT116 colon cancer, showed susceptibility to such bacteriocins [24].

In vitro studies conducted against various tissues and models have indicated that fermenticin HV6b produced by *Lactobacillus fermentum* HV6b MTCC 10770 has the potential to be used as a component of anticancer drug therapy because it has been reported to induce apoptosis in cancer cells [20].

The other bacteriocin, such as rec-pediocin CP2, has significantly higher cytotoxicity and chromosomal DNA damage in cell lines tested with bacteriocin [25].

### 5.5. Veterinary Use

The use of nisin as preventive medicine and as a remedy for mastitis in cattle has also been investigated in the veterinary industry [26]. Bovine mastitis is a disease that has a major economic impact in the global dairy industry, as it is the leading cause of economic loss among livestock farmers [7]. Nisin-based injectable drugs have been reported to control nearly 99.9% of bacteria that cause mastitis, such as *Staphylococcus aureus* and *Streptococcus agalactiae* after drug administration [26].

### 5.6. Skincare

Scientific and fact-based reports reinforce the assumption that certain probiotics may contribute to modulating the skin's microflora, lipid barrier, and skin's immune system, leading to the maintenance of skin homeostasis [27]. ESL5, a bacteriocin produced by *Enterococcus faecalis* SL-5, has been used as a lotion in a patient with inflammatory acne lesions caused by *Propionibacterium acnes*, which significantly reduces the inflammatory lesions and pimples compared to a placebo lotion [28].

### 5.7. Oral Care

*Lactobacillus plantarun* ACA-DC 269, *Lactobacillus fermentum* ACA-DC 179, and *Streptococcus macedonicus* ACA-DC 198 are the bacteria known to inhibit the growth of oral pathogens that cause oral health problems. Macedocin produced from *S. macedonicus* ACA-DC 198 is capable of killing oral pathogens in the lag phase by causing the main alterations in cellular chemistry that were confirmed by FTIR spectroscopy. Macedocin can be an active ingredient in formulations developed for toothpaste and mouthwash applications [29].

*Streptococcus mutans* cause dental caries leading to the decay of the teeth. Studies on nisin and polylysine showed that both compounds act on the same oral microbial flora. Partial inhibitory concentrations of nisin (200 IU) and polylysine (5 *μ*g/ml) delay bacterial growth when used separately. The combined use of them has a synergistic effect, inhibiting the bacterial growth for 24 h. A formulation containing 10 *μ*g/ml polylysine and 50 IU/ml nisin could completely inhibit *S. mutans*, 250 *μ*g/ml polylysine, and 100 IU/ml nisin eliminated total aerobic oral flora. This potential formulation has an application in the production of oral care products [30]. Halitosis is nothing but an oral malodor that is common in a few adult populations. It creates a significant problem in the social and working environment. Generally, this malodor is contributed by the growth of unwanted bacteria such as *Atopobium parvulum* ATCC33793, *Eubacterium sulci* ATCC35585, *Eubacterium saburreum* ATCC33271, *Parvimonas micra* ATCC33270, *Solobacterium moorei* CCUG39336, *Streptococcus anginosus* T-29, and *Micromonas micros*. *Streptococcus salivarius* K12 was isolated from the oral cavity of the healthy school going students. This strain produces 2 bacteriocins, salivaricin A2 and B. Salivaricins were found to be inhibitory against *Streptococcus pyogenes*, which causes pharyngitis [31]. Bacteriocins from *S. salivarius* K12 were able to inhibit the growth of all the abovedescribed bacteria causing halitosis. The development of novel formulations containing K12 strain, salivaricin, or a combination of both is worth considering [32].

### 5.8. Plant Growth Promotion

Different species of *Bacillus thuringiensis* were screened for the production of bacteriocins. *B. thuringiensis* NEB17 and BF4 produce bacteriocins thuricin 17 and bacthuricin F4, respectively. *Bacillus cereus* UW85 secretes bacteriocin C85. The molecular masses of these bacteriocins are between 3100 and 3200 Da, and these 3 bacteriocins could promote plant growth. A formulation containing the 3 bacteriocins and their producing bacteria was sprayed on tomato, corn, and soybean plants. The application of the formulation increased the leaf area, which led to a 6% increase in photosynthesis, a 15% increase in the dry weight of the plant, and a 21% increase in root nodulation in comparison with controls [33].

## 6. Conclusion and Prospects

Bacteriocins are widely known to have antimicrobial activities against pathogenic microorganisms. For this reason, the food and therapeutic applications of bacteriocins have gained increasing attention over the years. Bacteriocins have been proposed as a solution to the problems of food spoilage and food infections in the food industry. However, so far, the only commercially produced bacteriocins are nisin, produced by *Lactococcus lactis*, and pediocin PA-1, produced by *Pediococcus acidilactici*, marketed as NisaplinTM and ALTATM 2431, respectively. Luckily, there is an upward trend of both consumer preferences and the regulatory demand for minimally processed foods without the use of chemical preservatives. This offers a real opportunity that the widespread application of bacteriocins in the food industry could start.

On the other hand, the growing problem of multidrug-resistant pathogens and the rapidly declining antibiotic arsenal may be one of the greatest concerns facing humanity in the 21^st^ century. Researchers around the world are looking for possible alternatives to tackle this problem. The strong specific activity of certain bacteriocins against clinical pathogens, even against multidrug resistance strains, offers a possible solution to this growing problem. Thus, to further improve the bacteriocin arsenal against these unwanted spoilage microorganisms and pathogens, it is important to promote the study on the mode of antimicrobial action and their biosynthetic mechanisms of known bacteriocins to look for more new bacteriocins with promising properties.

## Figures and Tables

**Table 1:** Classification of bacteriocins adapted from [9].

Classification	Features	Subcategories	Examples
Class I bacteriocins (lantibiotics)	Lanthionine or peptides containing *β*-lanthionine	Type-A (linear molecules)	Nisin, subtilin, epidermine
		Type-B (globular molecules)	Mersacidin

Class II bacteriocins	Heterogeneous class of small thermostable peptides	Subclass IIa (antilisterial pediocine bacteriocins type)	Pediocin, enterocin, sakacin
		Subclass IIb (composed of two peptides)	Plantaricin, lactacin F
		Subclass IIc (other bacteriocins)	Lactococcin

Class III bacteriocins	Large thermolabile peptides		Helveticin J, millericin B

## Data Availability

The data used to support the findings of this article are available on request from the corresponding author.

## Abbreviations

AMPs: Antimicrobial proteins
BV: Bacterial vaginosis
Da: Dalton
FDA: Food and Drug Administration
FTIR: Fourier-transform infrared spectroscopy
GRAS: Generally recognized as safe
HNSCC: Head and neck squamous cell carcinoma
IU: International unit
kDa: Kilodalton
LAB: Lactic acid bacteria.
